# Effect of Preexisting Immunity to Tetanus Toxoid on the Efficacy of Tetanus Toxoid-Conjugated Heroin Vaccine in Mice

**DOI:** 10.3390/vaccines9060573

**Published:** 2021-06-01

**Authors:** Essie Komla, Oscar B. Torres, Rashmi Jalah, Agnieszka Sulima, Zoltan Beck, Carl R. Alving, Arthur E. Jacobson, Kenner C. Rice, Gary R. Matyas

**Affiliations:** 1 Laboratory of Adjuvant and Antigen Research, US Military HIV Research Program, Walter Reed Army Institute of Research, 503 Robert Grant Avenue, Silver Spring, MD 20910, USA; ekomla@hivresearch.org (E.K.); oscarbuenatorres@gmail.com (O.B.T.); rjalah@gmail.com (R.J.); zoltan.beck@gmail.com (Z.B.); calving@hivresearch.org (C.R.A.); 2 U.S. Military HIV Research Program, Henry M. Jackson Foundation for the Advancement of Military Medicine, 6720A Rockledge Drive, Bethesda, MD 20817, USA; 3 Drug Design and Synthesis Section, Molecular Targets and Medications Discovery Branch, Intramural Research Program, Department of Health and Human Services, National Institute on Drug Abuse and the National Institute on Alcohol Abuse and Alcoholism, National Institutes of Health, 9800 Medical Center Drive, Bethesda, MD 20892-3373, USA; agnieszka.sulima@nih.gov (A.S.); arthurj@nida.nih.gov (A.E.J.); kennerr@mail.nih.gov (K.C.R.)

**Keywords:** heroin vaccine, vaccine efficacy, conjugate vaccine, tetanus toxoid, preexisting immunity

## Abstract

Opioid use disorder (OUD) is a serious health problem that has dramatically increased over the last decade. Although current therapies for the management of OUD can be effective, they have limitations. The complementary strategy to combat the opioid crisis is the development of a conjugate vaccine to generate high affinity antibodies in order to neutralize opioids in circulation before reaching the brain. The components of an opioid vaccine include an opioid hapten (6-AmHap) that is conjugated to a carrier protein (tetanus toxoid) with the addition of adjuvants (Army Liposome Formulation adsorbed to aluminum hydroxide-ALFA). There is no consensus in the literature as to whether preexisting immunity to the carrier protein may impact the immunogenicity of the conjugate vaccine by inducing an enhanced or suppressed immune response to the hapten. Here, we investigated whether pre-exposure to tetanus toxoid would affect the immunogenicity and efficacy of the heroin vaccine, TT-6-AmHap. Mice were primed with diphtheria, tetanus, and acellular pertussis (DTaP) vaccine at weeks -4 and -2, then immunized with TT-6-AmHap vaccine at weeks 0, 3, and 6. Using ELISA and behavioral assays, we found that preexisting immunity to tetanus toxoid had no influence on the immunogenicity and efficacy of the TT-6-AmHap vaccine.

## 1. Introduction

Opioid use disorder (OUD) is a serious public health concern in the United States and around the world. The rate of opioid addiction and overdose-related deaths has increased exponentially over the last decade [1,2], with heroin and other synthetic opioids being the major contributors to the opioid crisis. In response to the opioid crisis, federal and state agencies have developed strategies to reduce the burden caused by opioid misuse through continued surveillance of medication-assisted treatment (MAT), which is the gold standard treatment for OUD [1,2,3]. Current FDA-approved medications for OUD include methadone, buprenorphine, naltrexone, in addition to naloxone, which is approved for opioid overdose reversal. Although these medications have been effective, their use comes with many limitations, including side effects (constipation, diarrhea, withdrawal symptoms) and compliance issues [3]. They have also failed to curb the continued growth of OUD in the US. An additional strategy that may be useful to combat the opioid crisis is vaccination against opioids.

Vaccines against drugs of abuse must induce high affinity antibodies that bind the drugs in the systemic circulation and prevent them from crossing the blood–brain barrier, thereby preventing the drug’s binding to and activation of the μ-opioid receptors (MOR) in the brain and their subsequent physiological effects. Opioids, such as heroin, are small molecules that lack the ability to elicit T-cell-dependent immune responses, which are characterized by immunological memory and potent antibody responses. To overcome this problem, a heroin hapten, 6-AmHap was developed. This hapten is conjugated to an immunogenic carrier protein, tetanus toxoid (TT), which can induce strong immune responses.

Tetanus toxoid (TT), diphtheria toxoid (DT) and cross-reacting material of diphtheria toxin with amino acid 197 substitution (CRM_197_) are three commonly used carrier proteins in licensed conjugate vaccines [4,5]. Multiple studies have reported that pre-immunization with homologous carrier proteins had positive or negative implications on the immunogenicity of the conjugate vaccines [6,7,8,9,10]. In one study, a single dose of HibV-TT in infants that were previously vaccinated with TT showed higher levels of protection against the *Haemophilus influenzae* type b (Hib) disease [11], whereas, in another study, priming with TT and DT in infants did not result in enhanced HibV-TT responses after three consecutive doses [12]. Similar discrepancies were also found with studies involving adults [13,14].

Since the majority of the population have been previously immunized with TT as part of the diphtheria, tetanus, and acellular pertussis (DTaP) vaccine or through continuing boosting with adult tetanus, diphtheria, and pertussis (Tdap) or TT alone, it is important to know if preexisting immunity to TT would impact the efficacy of the heroin vaccine. Here, we investigated whether priming with TT through administration of DTaP would affect the immunogenicity and efficacy of the heroin conjugate vaccine TT-6-AmHap in mice. Notably, our results revealed that antibodies generated to the priming agent, TT, had no impact on the ability of the TT-6-AmHap vaccine to induce high titer anti-hapten antibodies that blocked the effects of heroin.

## 2. Materials and Methods

### 2.1. Materials and Reagents

Liposomal lipids consisting of 1,2-dimyristoyl-*sn*-glycero-3-phosphocholine (DMPC), 1,2-dimyristoyl-*sn*-glycero-3-phosphoglycerol (DMPG), monophosphoryl lipid A (PHAD^®^), and semi-synthetic cholesterol were purchased from Avanti Polar Lipids (Alabaster, AL). Bovine Serum Albumin (BSA), which was used in the blocking buffer for ELISA. was purchased from Sigma-Aldrich (Saint Louis, MO). Heroin HCl was purchased from Lipomed Inc. (Cambridge, MA). Tetanus toxoid (TT) was purchased from Statens Serum Institut (Copenhagen, Denmark). DTaP (Infanrix^®^) was purchased from GlaxoSmithKline Biologicals (Rixensart, Belgium). BSA, SM(PEG)_2_ linker, and bicinchoninic acid (BCA) protein assay kit, which were used for the coupling reactions, were purchased from Pierce Protein Research/Thermo FisherScientific (Rockford, IL, USA). Immunolon 2HB flat-bottom ELISA plates were purchased from Thermo Scientific (Marietta, OH, USA). Diphtheria toxoid (DT), pertussis toxin (PT), and TT used as coating antigens for ELISA were purchased from List Biological Laboratories, Inc. (Campbell, CA, USA). Peroxidase-linked sheep anti-mouse IgG (γ-chain specific) was purchased from The Binding Site (San Diego, CA, USA). Peroxidase substrate solution was purchased from KPL, Inc. (Gaithersburg, MD, USA). Alhydrogel was purchased from Brenntag (Reading, PA, USA). BD Insulin Syringes (0.5 mL) used for vaccine injection were purchased from FisherScientific (Rockford, IL, USA). Pipettes and tips were purchased from Mettler and Toledo (Columbus, OH, USA).

### 2.2. Coupling Procedure

#### 2.2.1. TT Conjugate Antigen Synthesis

The heroin hapten, 6-AmHap was coupled to the tetanus toxoid (TT) carrier protein as described [15,16]. Briefly, TT was incubated with the SM-(PEG)_2_ cross-linker at a molar ratio of 1:1600 for 2 h at room temperature. Excess linker was removed by spin desalting column and the protein content was quantified by BCA protein assay. The hapten, 6-AmHap, was added dropwise to a stirring solution of TT−SM-(PEG)_2_ intermediate at a molar ratio of 1:300 and was incubated at room temperature for 2 h. Overnight dialysis against PBS, pH 7.4, at 4 °C was used to remove excess haptens. The TT−hapten conjugates were quantified using the BCA protein assay. The number of heroin haptens attached to the TT molecule was assessed by matrix-assisted laser desorption/ionization time-of-flight (MALDI-TOF) mass spectrometry (Supplementary Material, Figure S1). The 1:1600 TT:SM-(PEG)_2_ ratio yielded approximately 32 6-AmHap haptens per TT molecule.

#### 2.2.2. BSA-Hapten Conjugate Synthesis

BSA was coupled to 6-AmHap by incubating BSA and SM-(PEG)_2_ linker in PBS at a molar ratio of 1:10 for 2 h at room temperature. Excess linker was removed by spin desalting column. A 6-AmHap was added dropwise to a stirring solution of BSA−SM-(PEG)_2_ intermediate at a molar ratio of 1:300 and was incubated at room temperature for 2 h, followed by excess 6-AmHap removal by overnight dialysis against PBS, pH 7.4, at 4 °C. The BSA-6-AmHap conjugate was membrane filtered and quantified by BCA assay. The 1:10 BSA:SM-(PEG)_2_ ratio yielded approximately 5 haptens per molecule [16].

### 2.3. Vaccine Formulation and Immunization

#### 2.3.1. Vaccine Formulation

The formulation of TT-conjugated vaccine consisted of Army Liposome Formulation adsorbed to aluminum hydroxide (ALFA) and contained monophosphoryl lipid A (PHAD^®^) as adjuvant mixed with 6-AmHap conjugated to TT (TT-6-AmHap). ALFA was prepared as previously described [17,18]. In short, DMPC, cholesterol, DMPG, and PHAD^®^ were dissolved in solvents that were removed by rotary evaporation, hydrated in water and microfluidized. The molar ratio of DMPC:cholesterol:DMPG was 9:7.5:1, and the molar ratio of phospholipid:PHAD^®^ was 220:1. The multilamellar vesicles (MLVs) were lyophilized. The preformed multilamellar lyophilized liposomes contained 50 mM phospholipids which had been mixed 1:1 with TT-6-AmHap and then bound to Alhydrogel^®^ to give a final vaccine concentration of 10 μg TT, 30 μg aluminum hydroxide in Alhydrogel^®^, and 20 μg PHAD^®^ per dose of 50 μL in PBS, pH 7.4. The approximately 32 haptens/TT molecule ratio corresponds to approximately 0.8–0.9 µg of 6-AmHap/dose. The vaccine was made prior to each immunization. The TT-6-AmHap prepared in the engineering run for the GMP manufacture of TT-6-AmHap was stable at the most recent testing time point of 9 months from the date of manufacture.

#### 2.3.2. Animal Studies

Female BALB/c mice (*n =* 10 per group) (Jackson Laboratories, Bar Harbor, ME, USA) were immunized via the intramuscular (IM) route in alternate rear thighs at weeks -4 and -2 with 0.1 µL, 1 µL, 10 µL, or 25 µL of DTaP vaccine diluted in saline to a final volume of 50 µL for all groups. On weeks 0, 3, and 6, mice were immunized by the IM route in alternate rear thighs with 50 µL of TT-6-AmHap vaccine. DTaP doses were drawn using pipettes with varying sizes. Both DTaP and TT-6-AmHap vaccines were injected using 0.5 mL insulin syringes. All animals were bled prior to each immunization with TT-6-AmHap vaccine.

### 2.4. Behavioral Assays

#### 2.4.1. Hot Plate Test

Ten weeks after primary immunization with TT-6-AmHap, the animals were tested on a hot plate to assess the vaccine efficacy after heroin challenge. The test was performed following the protocol previously described [19]. Briefly, the hot plate was set to 56 °C. Baseline latency to the thermal stimulus was first assessed by placing mice on the hot plate (Harvard Apparatus, Holliston, MA, USA) and measuring the amount of time required until the mouse licked or lifted their hind paws or jumped. Following a subcutaneous injection with heroin (1 mg/kg heroin dissolved in saline), the mouse was assessed on the hot plate 15 min later. A cutoff time of 30 s was set to prevent burns. The hot plate data were quantified using the percentage of maximum possible effect (%MPE) equation: %MPE = [(challenge time − baseline time)/(cutoff time − baseline time)] × 100. Higher %MPE values indicated antinociception.

#### 2.4.2. Locomotion Test

Prior to the testing day, mice were acclimated to the locomotion chamber for 30 min on at least two occasions. The locomotion assay was performed immediately following the hot plate pre- and post-challenge tests. Baseline motor activity was recorded by an overhead camera (EverFocus^®^ Polestar II camera, New Taipei City, Taiwan) for 5 min. Following the subcutaneous administration of heroin (1 mg/kg), the mice were placed in the chamber and the movement monitored for 5 min. Data were analyzed using the EthoVision XT software (Noldus Information Technology Inc., Leesburg, VA, USA) to calculate the total distance traveled (cm) for each animal using the following equation: Total Distance Traveled (cm) = Distance _after heroin challenge_ − Distance _before heroin challenge_.

### 2.5. Immunogenicity Studies

#### Enzyme-Linked Immunosorbent Assay (ELISA)

Serum antibodies specific to 6-AmHap, TT, DT, and PT were assessed using ELISA. Plates were coated with BSA-6-AmHap, TT, DT or PT (0.1 μg/0.1 mL in PBS, pH 7.4) and incubated overnight at 4 °C. The ELISA was performed the next day as previously described [18]. Briefly, blocking buffer (1% BSA in 20 mM Tris-0.15 M sodium chloride, pH 7.4) was added to the plates and incubated at room temperature for 2 h. Sera were diluted in blocking buffer at 1:400 and added to the plates in triplicate. The sera were diluted serially down the rows of the plate. After 2 h of incubation at room temperature, the plates were washed with 20 mM Tris-0.15 M sodium chloride-0.05% Tween 20 and then peroxidase-linked sheep anti-mouse IgG was diluted in blocking buffer at 1:1000 and added to the plates for an incubation of 1 h at room temperature, followed by another wash. ABTS peroxidase substrate solution (100 μL/well) was added to the plates for 1 h at room temperature. The reaction was stopped with 1% sodium dodecyl sulfate (SDS), and the absorbance was read immediately at 405 nm.

### 2.6. Data Analysis

GraphPad Prism 8 (GraphPad Software, La Jolla, CA, USA) was used for statistical analyses. Antibody titers are expressed as endpoint titers which represent the dilution of sera where the absorbance is twice that found in the background. Data were analyzed using one- or two-way ANOVA as indicated in the figure legends. A post hoc test was performed using Tukey’s multiple comparisons test to identify differences between groups relative to the control. Differences were considered significant if *p* ≤ 0.05. All values represent the mean ± S.E.M.

## 3. Results

### 3.1. Effect of DTaP Immunization on the Immunogenicity of the TT-6-AmHap Vaccine

**Figure 1:**
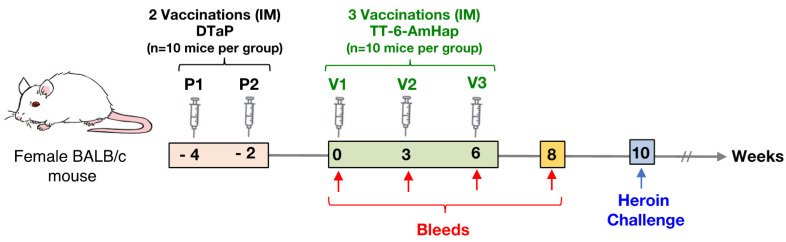
Study design for testing TT-6-AmHap vaccine efficacy in mice primed with DTaP. Groups of 10 mice were pre-immunized intramuscularly (IM) with increasing doses of DTaP (P1, P2) at weeks -4 and -2. Blood was collected from all mice prior to each vaccination with TT-6-AmHap. Mice were vaccinated with 10 μg TT-6-AmHap (V1, V2, V3) intramuscularly in alternate rear thighs at weeks at 0, 3, and 6. All mice, including heroin vaccine only (unprimed) and negative (naive) controls, were challenged with 1 mg/kg heroin SC at week 10. The hot plate test and locomotion assay were used to assess TT-6-AmHap vaccine efficacy in response to the heroin challenge.

**Figure 2:**
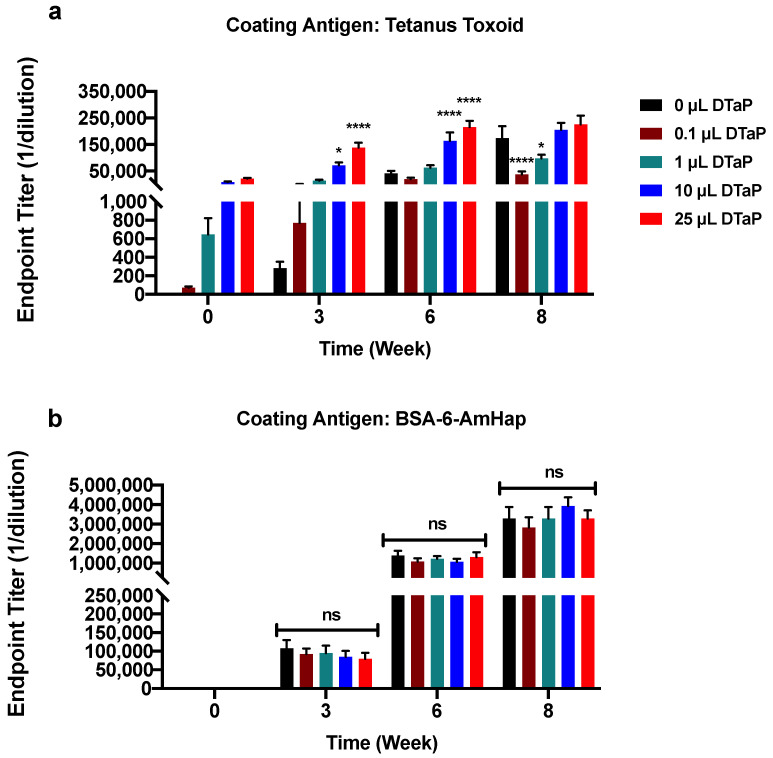
Serum IgG responses to tetanus toxoid and 6-AmHap from animals pre-immunized with DTaP. Antibody levels in response to (**a**) tetanus toxoid and (**b**) 6-AmHap in mice immunized with increasing doses of DTaP four weeks prior to primary immunization with TT-6-AmHap. Blood was collected from mice at weeks 0, 3, 6, and 8. Values represent the mean ± S.E.M. of triplicate determinations. Significance of endpoint titers between groups was determined by two-way ANOVA with Tukey’s multiple comparisons test. N = 10/group; * *p* < 0.05; **** *p* < 0.0001; ns: not significant; compared to 0 μL DTaP within the week.

**Figure 3:**
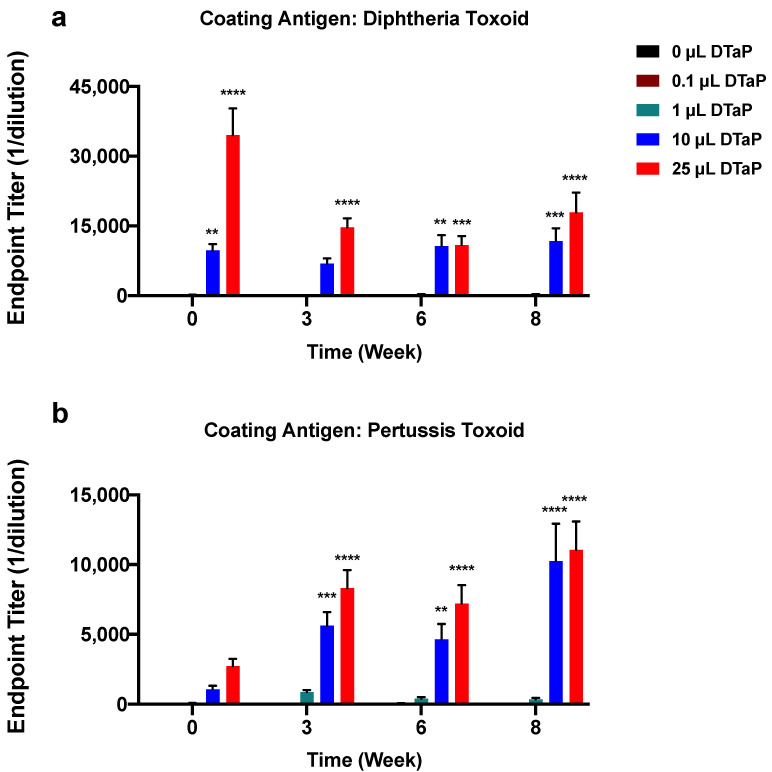
Serum IgG responses to diphtheria toxoid and pertussis toxoid in animals pre-immunized with DTaP. Antibody levels in response to (**a**) diphtheria toxoid and (**b**) pertussis toxoid in mice immunized with increasing doses of DTaP four weeks prior to primary immunization with TT-6-AmHap. Blood was collected from mice at weeks 0, 3, 6, and 8. Values represent the mean ± S.E.M. of triplicate determinations. Significance of endpoint titers between groups was determined by two-way ANOVA with Tukey’s multiple comparisons test. N = 10/group; ** *p* < 0.01, *** *p* < 0.001, **** *p* < 0.0001; compared to 0 μL DTaP within the week.

To determine if preexisting immunity to TT would interfere with TT-6-AmHap immune responses, mice were primed with increasing doses of the DTaP vaccine ranging from 1/5000th dose of DTaP (0.1 µL DTaP) to 1/20th dose of DTaP (25 µL DTaP) at weeks -4 and -2. Figure 1 illustrates the study design. After two immunizations (week 0) with DTaP, antibody levels of TT, DT, and PT were elevated for the 10 μL and 25 μL DTaP doses compared to control animals (0 µL DTaP), 0.1 µL DTaP, and 1 µL DTaP immunized animals (Figure 2a and Figure 3). Following immunization with TT-6-AmHap, the antibody titers to TT increased as a function of the priming dose of the DTaP vaccine (Figure 2a). Antibody titers to TT were significantly boosted after one dose of TT-6-AmHap (week 3) in the animals primed with the 1, 10, and 25 µL of DTaP. Following the second immunization with TT-6AmHap, the TT titers further increased in all animals, with the 10 and 25 µL DTaP-primed animals having the highest TT antibody titers. Following 3 doses of TT-6-AmHap (week 8), the TT antibody titers were high in the 0, 10, and 25 µL DTaP-primed groups. The animals primed with 0.1 and 1 µL DTaP had significantly lower TT antibody titers than the 0, 10, and 25 µL primed animals (Figure 2a).

Immunization with TT-6-AmHap induced high antibody titers to 6-AmHap. DTaP-primed animals had equivalent 6-AmHap antibody titers to those obtained from animals that received only the TT-6-AmHap vaccine (Figure 2b). Furthermore, after 2 priming doses of DTaP, antibody titers to DT were increased in mice primed with 10 and 25 µL DTaP compared to the mice primed with 0.1 and 1 µL DTaP at week 0. Mice primed with 25 µL DTaP had highly elevated DT titers at week 0, and then the titers decreased at week 3 and remained at the same level over weeks 6 and 8 (Figure 3a). Antibody titers to DT in mice primed with 10 µL DTaP, on the other hand, remained at the same level from week 0 to 8 (Figure 3a) and TT-6-AmHap did not further increase the antibody titers to DT. Moreover, DTaP priming immunization induced antibodies to PT in a dose-dependent manner with the 25 µL dose group having the highest titer from week 0 to 8 (Figure 3b). However, a significant PT antibody production was not observed until week 3 and remained stable through week 8 despite the presence of TT-6-AmHap. Consequently, priming with increasing doses of DTaP had no effect on the immunogenicity of the TT-6-AmHap heroin conjugate vaccine.

### 3.2. Efficacy of TT-6-AmHap Heroin Vaccine

**Figure 4:**
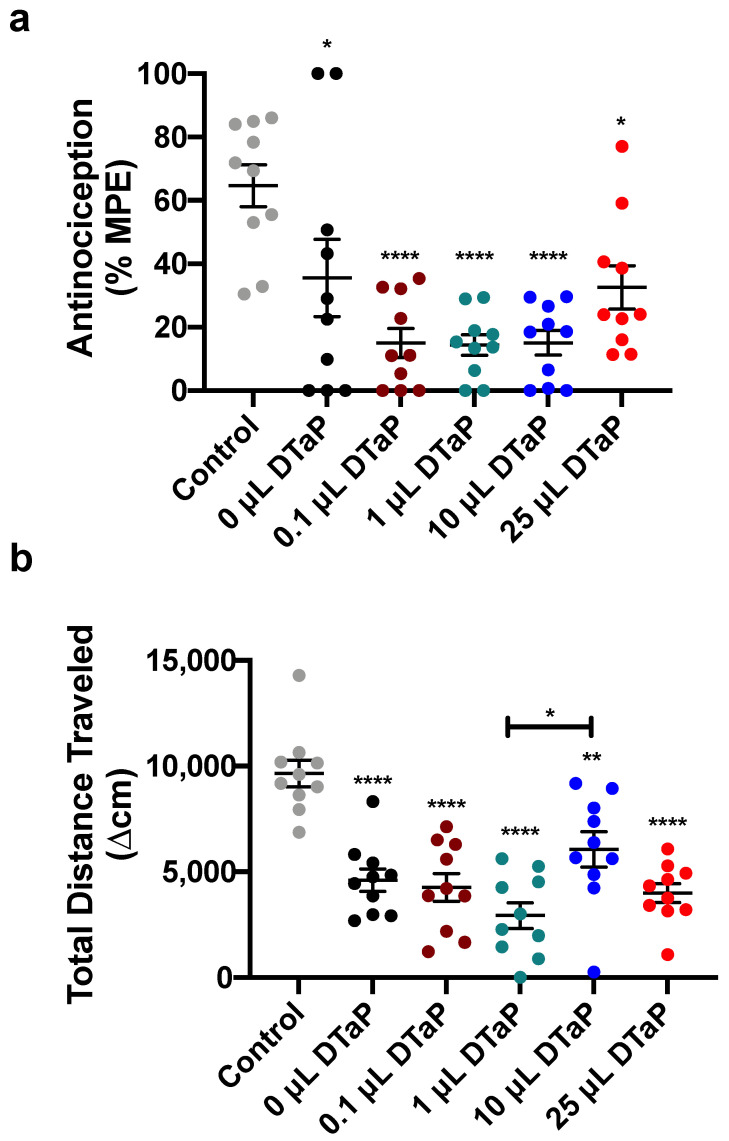
TT-6-AmHap vaccine efficacy in DTaP immunized animals as assessed on the hot plate and in the locomotion assays 10 weeks after primary immunization. Naive control mice (gray dots) and immunized mice were challenged with 1 mg/kg heroin SC and tested with the (**a**) hot plate nociceptive assay and (**b**) locomotion assay. Antinociception is expressed as percent maximum possible effect (%MPE) for the hot plate test. The difference between the pre- and post-heroin challenge values represents the total distance traveled (cm) in the locomotion assay. Values represent the mean ± S.E.M. Statistical differences between groups were determined by one-way ANOVA with Tukey’s multiple comparisons test. N = 10/group; * *p* < 0.05; ** *p* < 0.01; **** *p* < 0.0001; compared to the naive control; ns: not significant.

The efficacy of the TT-6-AmHap vaccine was tested by challenging the mice with 1 mg/kg heroin SC at week 10 followed by assessment using the hot plate and locomotion assays. Heroin-induced antinociception was significantly reduced in the TT-6-AmHap vaccine-only group and DTaP-primed groups compared to the naive control mice indicated by the low % maximum possible effect (% MPE) values (Figure 4a). There was no significant difference in the % MPE values of TT-6-AmHap vaccine-only group and DTaP-primed groups. Similarly, heroin-induced hyperlocomotion was significantly reduced in all the groups compared to the naive control mice (Figure 4b). There was no significant difference among the immunized animals except between the 1 and 10 µL DTaP-immunized animals. These results indicated that the TT-6-AmHap antibodies provided protection against the heroin challenge in mice despite the presence of TT, DT, and PT antibodies. In conjunction with the immunogenicity data, the vaccine efficacy data suggested that preexisting immunity to TT did not alter the efficacy of the heroin conjugate vaccine.

## 4. Discussion

In the present study, we sought to determine the effects of preexisting immunity to tetanus toxoid on the efficacy of a heroin-conjugated vaccine. Specifically, we determined the immunogenicity and efficacy of the TT-6-AmHap vaccine in mice pre-immunized with TT using ELISA and behavioral assays. We found that priming with increasing doses of DTaP did not interfere with the generation of hapten-specific antibodies, nor did it change the efficacy of the vaccine (Figure 2b and Figure 4). To the best of our knowledge, our study is the first to demonstrate the effect of preexisting immunity to the carrier protein (TT) on the efficacy of a conjugated drug of abuse vaccine (TT-6-AmHap). Most studies have focused on the effects of carrier priming on vaccines conjugated with polysaccharides [8,20,21] or bacterial/viral vectors [22,23,24]. Findings from these studies are rather confusing and contradictory as some have reported that priming with homologous proteins from the conjugate vaccines resulted in an enhancement of hapten-specific antibodies [8,9], while others have reported a suppression [10,25].

We found that priming with TT through DTaP administration enhanced TT immune responses in the vaccinated animals with TT-6-AmHap (Figure 2a), demonstrating an additive effect. This was shown at week 0 before the heroin vaccine immunization and at subsequent weeks (3, 6, 8) after, indicating a dose- and time-dependent relationship between the priming agent and the carrier protein. Similar to the TT immune responses at week 0, DT antibody levels were initially increased as a result of the highest dose of DTaP immunization. That level decreased soon after and continued to decrease over the following weeks and then remained stable, mimicking a normal immune response after an antigen stimulation. PT antibody titers, on the other hand, were very low after the priming doses at week 0, then progressively increased time-dependently and remained stable over the course of the weeks. One possibility for the cause of the low PT antibody titers is that they are slow to develop, reaching maximal titer several weeks after DTaP boosting.

While others have reported that preexisting immunity to the priming agent suppressed hapten-specific antibody responses [20,26], we have found that the presence of antibodies and presumably B-cell memory to TT, DT, and PT had no effect on the immunogenicity of the TT-6-AmHap vaccine (Figure 2b). The antibodies generated to 6-AmHap in the heroin vaccine-only control group (0 μL DTaP) were consistent with our previous work [27]. Our findings were quite intriguing considering the controversy surrounding the “carrier priming” and the “carrier-induced epitope suppression” phenomenon. A number of studies have found either an enhancement or suppression of the immune response to the conjugate vaccine [8,25,28]. For example, in one study the antibody response to PCV-TT and MenCV-TT was enhanced in pre-immunized mice with TT [21]. However, the authors concluded that priming with low dose TT (0.025–0.25 μg) resulted in an enhanced antibody response, whereas a high dose of TT (25 μg) resulted in a suppressing effect [21]. We did not observe any suppressing effects even though the highest dose of DTaP used in our study was 25 μL, which contained 1.5 μg TT. Conversely, others have found an enhancement with higher doses of the priming agent [5,29,30].

These conflicting findings in the literature could be due to several factors, including the type of carrier protein used, the concentration of the carrier protein, the hapten–carrier molar ratio, and the choice of adjuvants. Our heroin vaccine contained 10 μg of TT as a carrier with approximately 32 6-AmHap molecules per TT and was adjuvanted with liposomes containing monophosphoryl lipid A (PHAD^®^) and aluminum hydroxide. We have previously demonstrated the superiority of TT over CRM_197_ conjugate vaccines in terms of inducing potent inhibition of heroin-induced antinociception [15]. Furthermore, we have also shown that increasing the hapten density of the TT created a steric hindrance, thus decreasing the immunogenicity of the carrier protein [15,16]. Others have found similar findings [29,31]. Our findings, along with others, suggest that the ideal carrier protein should be the one which induces a potent immune response to the hapten instead of itself [8].

In addition to not having any influence on anti-6-AmHap immune responses, pre-immunization with TT did not impact the efficacy of the heroin vaccine. The vaccine showed an equal protection against the heroin challenge in the antinociceptive and the locomotion assays in all groups (Figure 4). Although there was a slight increase in the 25 µL DTaP group in the antinociception assay in 2 out of 10 mice, there was no statistical difference among the vaccinated groups. In the same fashion, there was a slightly significant difference (*p* = 0.011) between the 1 µL and 10 µL DTaP groups in the locomotion assay (Figure 4b); however, the heroin-induced hyperlocomotion was still reduced in these two groups compared to the control animals. Findings from the efficacy studies support our previously published work [27] as they indicated the presence and drug-binding capacity of hapten-specific antibodies in circulation and revealed the propensity for protection against the heroin challenge.

## 5. Conclusions

Preexisting immunity to tetanus toxoid had no effect on the antibody titer or efficacy from the heroin challenge induced by immunization with the 6-AmHap heroin vaccine. Neither an enhancement nor a suppressive effect on the efficacy of the TT-6-AmHap conjugate vaccine was observed. Considering most people have a preexisting immunity to TT, the data from this study suggest that this will not reduce the efficacy of the TT-6-AmHap heroin vaccine.

## Data Availability

Data presented in this study are available from the corresponding author upon request.

## Supplementary Materials

The following are available online at https://www.mdpi.com/article/10.3390/vaccines9060573/s1, Figure S1: MALDI-TOF MS spectra of protein antigens.
