# Supplementary material for: Effect of Preexisting Immunity to Tetanus Toxoid on the Efficacy of Tetanus Toxoid-Conjugated Heroin Vaccine in Mice
Source: Vaccines (Basel). 2021 Jun 1;9(6):573. doi: 10.3390/vaccines9060573 (PMC8229309; doi:10.3390/vaccines9060573)
Supplement: Supplementary file 1 [file vaccines-09-00573-s001.zip › vaccines-1219585-supplementary.pdf]

Supplementary material

Effect of preexisting immunity to tetanus toxoid on the efficacy of tetanus toxoid-conjugated heroin vaccine in mice

Essie Komla[1,2], Oscar B. Torres[1,2,a], Rashmi Jalah[1,2,b], Agnieszka Sulima[3], Zoltan Beck[1,2,c], Carl R. Alving[1], Arthur E. Jacobson[3], Kenner C. Rice[3], Gary R. Matyas[1,*]

[1] Laboratory of Adjuvant and Antigen Research, US Military HIV Research Program, Walter Reed Army Institute of Research, 503 Robert Grant Avenue, Silver Spring, Maryland 20910, United States; ekomla@hivresearch.org (E.K.); calving@hivresearch.org (C.R.A.)

[2] U.S. Military HIV Research Program, Henry M. Jackson Foundation for the Advancement of Military Medicine, 6720A Rockledge Drive, Bethesda, Maryland 20817, United States.

[3] Drug Design and Synthesis Section, Molecular Targets and Medications Discovery Branch, Intramural Research Program, National Institute on Drug Abuse and the National Institute on Alcohol Abuse and Alcoholism, National Institutes of Health, Department of Health and Human Services, 9800 Medical Center Drive, Bethesda, Maryland 20892-3373, United States; agnieszka.sulima@nih.gov (A.S.); arthurj@nida.nih.gov (A.E.J); kennerr@mail.nih.gov (K.C.R)

[a] Current address: Merck & Co., Inc., 126 E Lincoln Avenue, Rahway, New Jersey 07065, United States; oscarbuenatorres@gmail.com

[b] Current address: GlaxoSmithKline Vaccines, 14200 Shady Grove Rd, Rockville, Maryland 20850, United States; rjalah@gmail.com

[c] Current address: Pfizer, 401 N Middletown Rd, Pearl River, New York 10965, United States; zoltan.beck@gmail.com

* Correspondence: gmatyas@hivresearch.org; Tel.: 301-319-9973

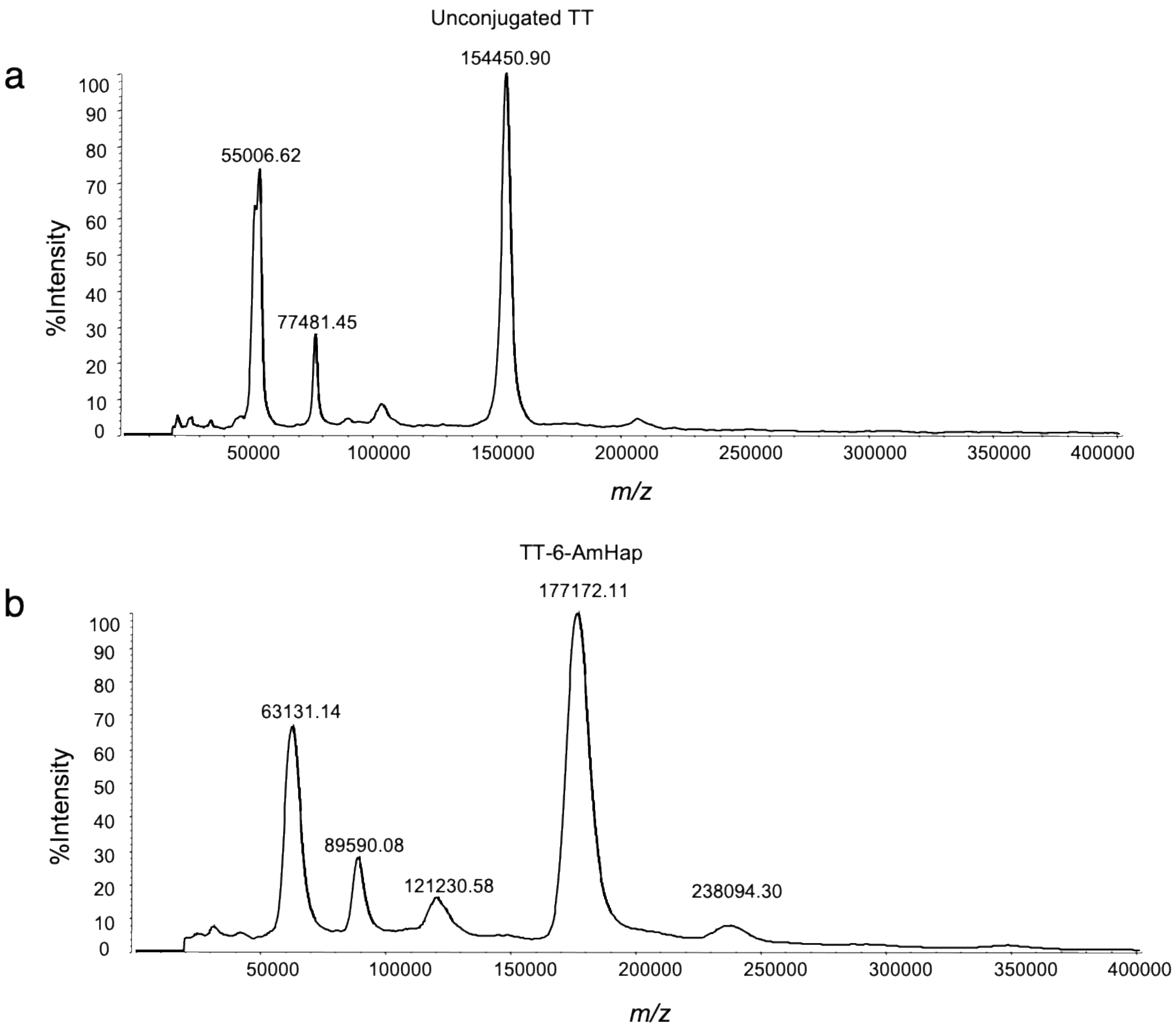

Figure S1: MALDI-TOF MS spectra of protein antigens. Spectra of (a) unconjugated TT carrier protein and (b) TT-6-AmHap conjugate. The instrument was calibrated using BSA as standard. Gaussian method was used to smooth the spectra and threshold apex peak detection method was used to assign masses.
